# Characterization of Biofilm Formation by *Mycobacterium chimaera* on Medical Device Materials

**DOI:** 10.3389/fmicb.2020.586657

**Published:** 2021-01-11

**Authors:** Archana D. Siddam, Shari J. Zaslow, Yi Wang, K. Scott Phillips, Matthew D. Silverman, Patrick M. Regan, Jayaleka J. Amarasinghe

**Affiliations:** ^1^Winchester Engineering and Analytical Center, United States Food and Drug Administration, Winchester, MA, United States; ^2^Center for Devices and Radiological Health, Food and Drug Administration, Silver Spring, MD, United States

**Keywords:** *Mycobacterium chimaera*, NTM, biofilm, heater-cooler devices, HCDS

## Abstract

Non-tuberculous mycobacteria (NTM) are widespread in the environment and are a public health concern due to their resistance to antimicrobial agents. The colonization of surgical heater-cooler devices (HCDs) by the slow-growing NTM species *Mycobacterium chimaera* has recently been linked to multiple invasive infections in patients worldwide. The resistance of *M. chimaera* to antimicrobials may be aided by a protective biofilm matrix of extracellular polymeric substances (EPS). This study explored the hypothesis that *M. chimaera* can form biofilms on medically relevant materials. Several *M. chimaera* strains, including two HCD isolates, were used to inoculate a panel of medical device materials. *M. chimaera* colonization of the surfaces was monitored for 6 weeks. *M. chimaera* formed a robust biofilm at the air-liquid interface of borosilicate glass tubes, which increased in mass over time. *M. chimaera* was observed by 3D Laser Scanning Microscopy to have motility during colonization, and form biofilms on stainless steel, titanium, silicone and polystyrene surfaces during the first week of inoculation. Scanning electron microscopy (SEM) of *M. chimaera* biofilms after 4 weeks of inoculation showed that *M. chimaera* cells were enclosed entirely in extracellular material, while cryo-preserved SEM samples further revealed that an ultrastructural component of the EPS matrix was a tangled mesh of 3D fiber-like projections connecting cells. Considering that slow-growing *M. chimaera* typically has culture times on the order of weeks, the microscopically observed ability to rapidly colonize stainless steel and titanium surfaces in as little as 24 h after inoculation is uncharacteristic. The insights that this study provides into *M. chimaera* colonization and biofilm formation of medical device materials are a significant advance in our fundamental understanding of *M. chimaera* surface interactions and have important implications for research into novel antimicrobial materials, designs and other approaches to help reduce the risk of infection.

## Introduction

*Mycobacterium chimaera* is a slow-growing non-tuberculous mycobacterial (NTM) species ubiquitously found in the environment, including tap water (Falkinham, 2009; Wallace et al., 2013; Glickman et al., 2020). *M. chimaera* was first identified as a part of the *Mycobacterium avium* complex (MAC) in 2004 (Tortoli et al., 2004). It is previously reported as an opportunistic respiratory pathogen associated with pulmonary diseases in immunocompromised individuals (Tortoli et al., 2004; Cohen-Bacrie et al., 2011). Recently, *M*. *chimaera* has been identified as a causative agent of invasive infections in patients undergoing cardiothoracic surgeries worldwide (Achermann et al., 2013; Sax et al., 2015). It was determined that heater cooler devices (HCDs) used to thermoregulate patients’ body temperature during surgery were colonized with *M. chimaera*, and that bioaerosols containing *M. chimaera* released from HCD during surgery may have led to the air-born transmission of these emerging pathogens to the patient (FDA, 2015, 2016, 2020a; Sommerstein et al., 2016, 2017; Chand et al., 2017).

HCDs consist of water tanks that deliver temperature-controlled water via closed circuits to a patient warming blanket, oxygenator, and/or cardioplegia solution, respectively (Sarkar and Prabhu, 2017; FDA, 2020a). Although the water in HCD circuits is not intended to be in direct contact with the patient or the patient’s blood, some studies have suggested that the gaps at the top of the water tanks and a series of holes near the water flow and return pipes of the water circuits can act as escape routes of aerosols containing *M. chimaera* from colonized HCDs (Sommerstein et al., 2016; Chand et al., 2017). HCD’s operational temperature range is between 2 and 41°C and standby units are kept at room temperature, a condition that may promote microbial build-up in both water tanks and connected tubes if maintenance and disinfection are performed poorly (FDA, 2016, 2020a; Sommerstein et al., 2016). Fans present in these devices may also facilitate the movement of aerosolized bacteria into the sterile surgical field. A simulated operating room smoke test showed that air exhausted from an HCD can reach the patient if the device is oriented with the fan facing toward the surgical field and within a short distance of the operating table (Sommerstein et al., 2016).

NTM biofilms can withstand a wide range of temperatures, pH, and low nutrient starvation conditions—posing a challenge to the successful disinfection (Esteban and Garcia-Coca, 2017). Biofilms are defined as communities of microbes living attached to surfaces (Costerton, 1995; Costerton et al., 1995). Bacteria that reside in a biofilm are embedded in a protective matrix called extracellular polymeric substance (EPS) that acts as a barrier against microbial agents and inhibits conventional disinfection strategies (Costerton et al., 1995, 1999; Donlan and Costerton, 2002). In work from 2016, it was found to be challenging to completely remove all *M. chimaera* biofilm from colonized HCDs (Schreiber et al., 2016; Falkinham, 2020). Some observed resistance to disinfectants may be due to several factors, including the ability of *M. chimaera* to form biofilms. While intensified disinfection protocols developed in response can improve planktonic microbicidal effectiveness, understanding and addressing biofilm formation by this emerging pathogen is important. There is a challenging tradeoff between increasing the concentration of antimicrobials and the potential for corrosion of metal components (Garvey et al., 2017, 2016). Some mycobacteria can also enter metabolic dormancy during nutrient and oxygen limitation, develop antimicrobial resistance and reemerge after an inactive period (Archuleta et al., 2005; Falkinham, 2018). The abundance of mycolic acids in mycobacterial cell walls form a hydrophobic extracellular matrix which acts as a permeability barrier to antibiotics and antimicrobial agents, resulting in the antimicrobial-resistant phenotype observed in NTM species (Esteban and Garcia-Coca, 2017). Horizontal gene transfer in NTM biofilms may also increase the development of resistance to standard antimicrobial agents and antibiotics (Le Dantec et al., 2002; Bryers, 2008; Huitt and Daley, 2015).

In addition, other water-bearing medical devices, including poorly maintained dental unit waterlines and thermoregulatory devices used in extracorporeal circuit membrane oxygenation (ECMO) have been shown to be colonized with various NTM, including *M. chimaera* (Dutil et al., 2007; Barbot et al., 2012; FDA, 2015, 2016, 2020a; Trudzinski et al., 2016; Chand et al., 2017). NTM contamination of medical devices can occur from multiple sources (Haller et al., 2016; Nishiuchi et al., 2017; van Ingen et al., 2017; Schreiber et al., 2018; Hasan et al., 2019). Therefore, the risk of NTM infection is a concern throughout medicine and especially for HCDs.

To develop strategies to eradicate *M. chimaera* from medical device surfaces, there is a need to better understand how these bacteria form biofilms on these materials, including the types of materials that they can colonize and the dynamic, temporal and morphological characteristics of these biofilms. To-date characterization of these biofilms has been limited due to the slow-growing nature of NTM. Herein, we report the ability of *M. chimaera* to attach and accumulate on a panel of commonly used medical device materials including stainless steel, titanium, silicone and polystyrene surfaces.

The study used multiple *M. chimaera* clinical strains including HCD isolates to demonstrate the ability of *M. chimaera* to form biofilms on various medical device materials in media as well as in autoclaved tap water at 30°C. First the formation of biofilm at the air-liquid interface in glass tubes was investigated since pellicle biofilm formation is a well-known behavior of NTM. Next, *M. chimaera* biofilm accumulation overtime was measured by a standard microtiter peg plate-based CV binding assay. Next, various medical device materials were inoculated with NTM species including *M. chimaera* and observed by 3D Laser scanning microscopy and electron microcopy at different timepoints up to 6 weeks. Finally, the biofilms formed on these materials were studied in more detail using cryo-SEM, allowing for preservation of ultrastructural components within the biofilm. Several unexpected and important discoveries about *M. chimaera* colonization are detailed and the implications for cleaning and disinfection are considered.

## Materials and Methods

### Bacterial Strains, Media, and Culture Conditions

All bacterial species used in this study are listed in Table 1 and were first cultured from frozen stocks stored at −80°C in 25% (w/v) glycerol to Middlebrook 7H10 agar (Difco) supplemented with 10% Middlebrook OADC enrichment (Difco) and 0.4% glycerol and incubated for 2 weeks at 30°C. Three to five well-isolated colonies were then used to inoculate 50 mL of sterile Middlebrook 7H9 broth (Difco) supplemented with 10% Middlebrook ADC enrichment (Difco) and 0.2% glycerol in a 250 mL Erlenmeyer flask and incubated aerobically at 30°C with shaking at 150 rpm for a week. Immediately before starting assays, the turbidity of the cultures was measured at optical density of 595 nm (OD_595_) using a spectrophotometer and diluted to an OD_595_ of 0.05 (approximately 10^7^ CFU/mL) in either 7H9 medium or autoclaved tap water for each species. The cultures were then, grown statically without replacement of media at 30°C for the duration required by the assay.

**TABLE 1:** Bacteria used in this study.

Bacterial Species	Description	Source
*Mycobacterium chimaera* (DSM 44623)	Human bronchial lavage isolate	Leibniz-Institut DSMZ
*Mycobacterium chimaera* (2015-22-08-01)	Pennsylvania HCD isolate	Center for Disease Control
*Mycobacterium chimaera* (2016-20-02)	North Dakota HCD isolate	Center for Disease Control
*Mycobacterium fortuitum* (ATCC 6841)	Fast Growing NTM	American Type Culture Collection

### Crystal Violet-Based Biofilm Assays and Quantification

A standard biofilm crystal violet staining assay on borosilicate glass tubes or 96-well polystyrene microtiter peg plates (Thermo fisher Nunc-TSP) were carried out as previously described with minor modifications (Amarasinghe et al., 2013). Briefly, exponentially growing *M. chimaera* DSM 44623 cultures were diluted to an OD_595_ of 0.05 in 7H9 media, as mentioned above prior to inoculation of borosilicate glass tubes (3 mL final volume) or 96-well polystyrene microtiter peg plates (200 μl per well) and, incubated statically with no disturbance at 30°C for up to 6 weeks. Biofilm attachment to borosilicate glass tubes was determined by performing CV binding assays at 6 weeks. Briefly, at the end of 6 weeks incubation, culture supernatants were discarded, and glass tubes were washed with PBS (pH 7.4) to remove any unattached cells. Next, 0.1% crystal violet dye (Sigma-Aldrich Co.) was added to borosilicate glass tubes and left at room temperature for 30 min. The unbound dye was then discarded, and the tubes were washed with tap water and digitally photographed.

For polystyrene PEG plate-based biofilm quantification assays, plates with cultures (200 μl per well) were placed in sterile plastic bags with wet towels to provide humidity and incubated at 30°C statically for 3 or 6 weeks without replacing the media. Media only wells/pegs were included as a negative control for each assay. CV staining was performed as described above, on both peg lid and 96 well plate bottom. Culture supernatants were decanted, and unbound bacteria were removed by washing with PBS (pH 7.4). The remaining cells and cell-associated materials were stained with CV for 30 min. Dye was then decanted, and wells were washed with tap water until negative control wells were clear. Stained cells and cell-associated materials were solubilized with ethanol. Absorbance was quantified at 570 nm. Each experiment has been done in triplicate and three biologically independent tests were used to measure the average biofilm accumulation. Student *t*-Test was performed to identify the statistical significance between the biofilm formed on pegs incubated with *M. chimaera* and the control pegs.

### 3D Laser Scanning Microscopy (3DLSM)

3D Laser scanning microscopy was performed to examine the attachment and thickness of *M. chimaera* biofilm on various medical device surfaces. Custom made (width-25 mm and length-75 mm) stainless steel, titanium, silicone and polystyrene coupons (BioSurface Technologies, Bozeman, MT, United States) were sterilized and placed into separate sterile Petri dishes. Exponentially grown *M. chimaera* cells, were diluted in 7H9 broth to an OD_595_ of 0.05, and then added to the Petri dish completely covering the coupon and incubated at 30°C statically for 1 week. After a week of incubation, the bacterial planktonic culture was carefully extracted without disturbing the biofilm containing coupon. Then the coupons were removed and analyzed under a 3D Laser Scanning Microscopy (3DLSM, Keyence model VK-X200). *Mycobacterium fortuitum*, a fast-growing NTM species, was cultured and imaged under similar conditions as *M. chimaera* and used as a positive control, but not as a surrogate for *M. chimaera.*

### Field Emission Scanning Electron Microscopy (FE-SEM)

*M. chimaera* biofilm structural analysis was performed using FE-SEM. For this, exponentially growing *M. chimaera* cells were diluted in either autoclaved tap water or 7H9 medium to an OD_595_ of 0.05 as described in culture conditions above and added to six separate 96-well polystyrene peg plates (200 μl per well) or 12-well tissue culture plates containing stainless steel or titanium coupons covering the coupon and carefully placed in sterile plastic bags with wet towels to provide humidity and incubated at 30°C statically for 1–6 weeks as required by the protocol. A row of wells with media alone were included with each polystyrene plate as the negative control. Additional stainless steel or titanium coupons with 7H9 or autoclaved tap water alone were used as the negative controls throughout this study. Each week, biofilm grown on pegs (from separate plates, up to 6 weeks) or coupons (at 2 weeks of incubation) was extracted from the culture medium, gently washed with PBS, and fixed in 3% glutaraldehyde in 0.1 M phosphate buffer (pH 7.3) for overnight at 4°C. Coupons were washed three times in 0.1 M sodium phosphate buffer (pH 7.3) for 10 min each. Samples were dehydrated using an ethanol dilution series (25, 50, 70, and 100%), performing each step twice for 10 min with a final step of three times for 15 min each. Samples were then chemically dried with a series of hexamethyldisilazane (HMDS) diluted in ethanol (ethanol:HMDS; 2:1, 1:1, 1:2) for 10 min each with a final wash in 100% HMDS twice. A second wash of HMDS was left to dry under the hood overnight. Samples were mounted onto the SEM sample holder using double-sided carbon tape and sputter-coated with Iridium for 30 s. Coated samples were imaged at various magnifications with a primary electron beam of 1 keV using a field emission scanning electron microscope (Supra 55VP, Zeiss, Thornwood, NY, United States) at the Image and Chemical Analysis Laboratory located at the Montana State University.

### Cryo-Scanning Electron Microscopy (Cryo-SEM)

Exponentially growing *M. chimaera* cultures from strains DSM 44623, 2015-22-08-01 and 2016-20-were diluted in autoclaved tap water to an OD_595_ of 0.05 and inoculated sterile 12-well tissue culture plates containing stainless steel and titanium coupons grown statically at 30°C for 24 h or 2 weeks as requirement by the assay before sample preparation. Biofilm grown coupons were removed from the culture medium and gently washed with PBS to remove the unattached cells. To rapidly cool the samples, coupons were carefully submerged into liquid nitrogen for 1–2 min and stored at −80°C until ready to image. Before imaging, samples were taken out, re-submerged in liquid nitrogen to keep frozen, and then loaded into the FE-cryo SEM stage. After loading to the stage, the temperature was set to −80°C (vapor pressure of ice) to sublimate the ice on the samples and the stage. After sublimation, the temperature was set to −193°C and images were taken with a primary electron beam energy of 1 keV at various magnifications.

## Results

### Quantification of *M. chimaera* Biofilm

**FIGURE 1:**
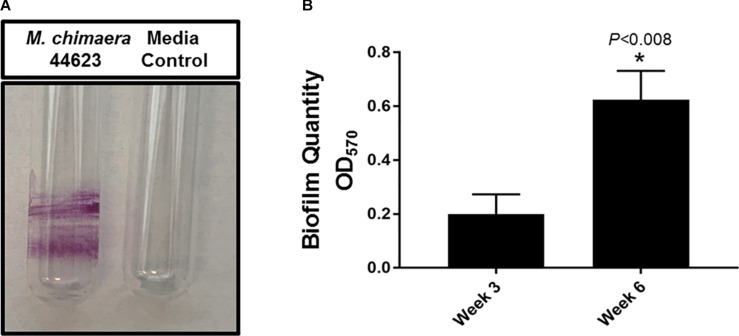
*Mycobacterium chimaera* biofilm quantification. **(A)** Crystal violet (CV) staining of 6-week old *M. chimaera* (DSM 44623) biofilm formed on borosilicate glass tube compared to the media control, **(B)** comparison of *M. chimaera* biofilm quantity on polystyrene PEG surface between week 3 and week 6.

The biofilm-forming capacity of *M. chimaera* was determined with CV staining. *M. chimaera* formed a biofilm at the air-liquid interface as compared to the media control on borosilicate glass tubes during 6 weeks of incubation at 30°C (Figure 1A). *M. chimaera* biofilm was also quantified using colorimetric CV assay. The optical density (OD) value for CV staining presented in Figure 1B is roughly proportional to the amount of biofilm extracted from the surface. Biofilm grown on polystyrene pegs subjected to CV staining and elution showed significantly greater amount of biomass produced by *M. chimaera* at week 6 compared to the amount at week 3 (Figure 1B). *M. chimaera* biofilm accumulation on pegs was found to be a slow but continuous process over 6 weeks.

### *M. chimaera* Forms Biofilm on Medical Device Material

**FIGURE 2:**
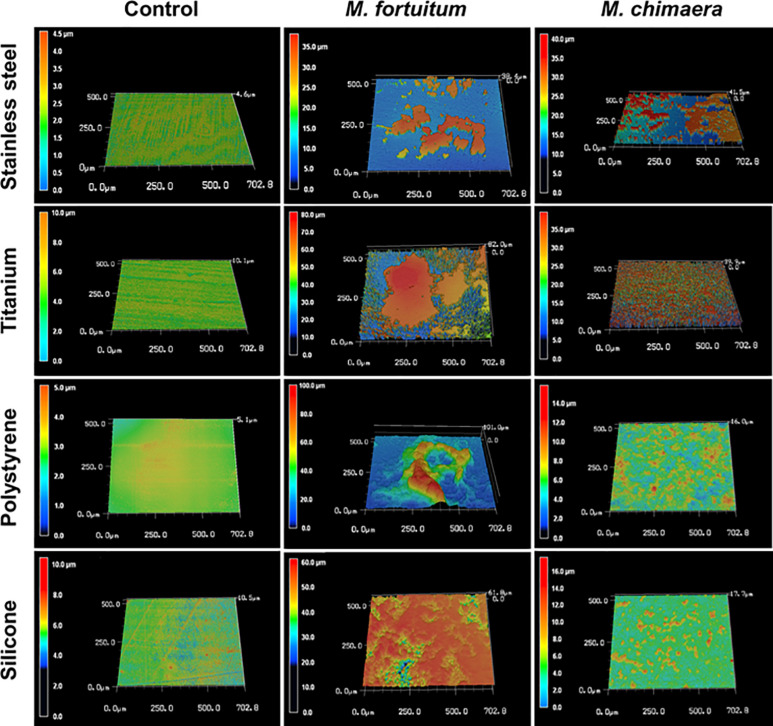
*Mycobacterium chimaera* DSM 44623 forms robust biofilms on various medical device surfaces. 3DLSM *analysis revealed that M. chimaera* attaches and forms a robust biofilm on both the stainless steel and titanium surfaces compared to the polystyrene or silicone surfaces within a week of incubation at 30°C **(right panel)**. A fast-growing NTM species, *M. fortuitum* was used as a positive control similar growth condition **(middle panel)**. Stainless steel, titanium, polystyrene, or silicone coupons incubated with media only were used as negative controls **(left panel)**.

Four common medical device materials (Stainless-steel, titanium, polystyrene, and silicon) showed differing biofilm coverage and morphology when visualized by 3DLSM at 1 week of incubation (Figure 2). Most of the control material samples, as expected, were smooth with minimal surface features (Figure 2, left panel). *M. fortuitum* formed thick biofilms covering large areas of the surface on all materials. Stainless steel had the least surface coverage of biofilm (Figure 2, middle panel-stainless steel), while silicone was nearly completely covered (Figure 2, middle panel-silicon). However, polystyrene, which had similar amounts of surface coverage to stainless steel, had the thickest biofilm (101 micron), while titanium had intermediate surface coverage with some areas reaching closer the thickness of 82 microns (Figure 2, middle panel-polystyrene or titanium). For *M. chimaera*, as expected due to its slower replication rate than *M. fortuitum*, biofilms observed at 1 week generally covered less of the surface and were thinner than those seen for *M. fortuitum* (Figure 2, right panel). Stainless steel had the thickest biofilm and about half of the surface appeared to be covered, while titanium had uniform surface coverage of a thinner biofilm (Figure 2, right panel-stainless steel or titanium). Both of the plastic surfaces had what appeared to be scattered, small emerging colonies that had not achieved continuous biofilm coverage as on the metal surfaces (Figure 2, right panel-polystyrene or silicon). Therefore, significant differences were observed in the materials preference between the two NTM, *M. fortuitum* and *M. chimaera*. *M. chimaera* preferentially attached to stainless steel and titanium surfaces in contrast to polystyrene and silicone surface (Figure 2, right panel) while the fast-growing *M. fortuitum*, formed thicker biofilms with different preferences (Figure 2, middle panel).

### *M. chimaera* Colonization and Biofilm Visualization Using Conventional Field Emission Scanning Electron Microscopy (FE-SEM)

**FIGURE 3:**
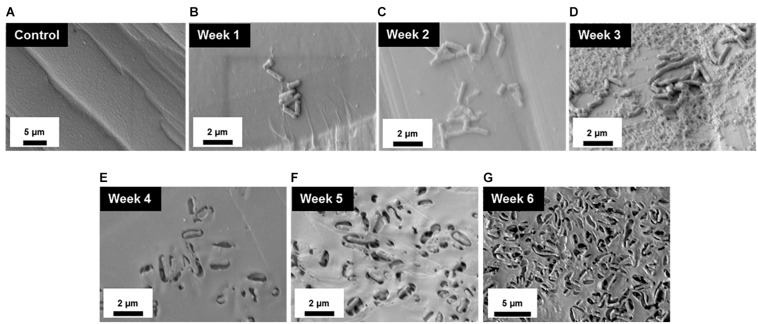
*Mycobacterium chimaera* cells became completely embedded in EPS on polystyrene surfaces by 6 weeks. Structural analysis of *M. chimaera* biofilms was performed using FE-SEM. *M. chimaera* (DSM 44623) biofilm grown on a polystyrene surface was collected from week 1 to week 6. **(A)** Polystyrene pegs incubated with media alone were used as the negative control for the experiment over 6 weeks. **(B)** At week 1, a small number of cells have been observed to be attached to peg surface. **(C)** At week 2, small aggregates of cells continued to spread on the surface. **(D)** By week 3, EPS matrix formation was observed **(E–G)**. From week 4 to week 6, these cells were entirely covered by the EPS material.

To better understand the temporal and morphological nature of increased *M. chimaera* biofilm formation on stainless steel and titanium surfaces, an in-depth structural analysis of these biofilms was performed using conventional field emission scanning electron microscopy (FE-SEM). For the initial investigation, *M. chimaera* biofilms grown in 7H9 media on polystyrene peg surfaces were collected at multiple maturation stages from week 1 to week 6; and analyzed for its attachment and EPS formation. At week 1, *M. chimaera* biofilm attached to the polystyrene surface minimally with increases in EPS production and colonization over time (Figures 3B–G). At week 2, *M. chimaera* cells continue to spread on the polystyrene surface (Figure 3C), and by week 3, these cells appeared to secrete extracellular material (Figure 3D). By week 6, these cells were entirely covered by the extracellular material (Figures 3E–G). Polystyrene peg surfaces with media alone were used as negative controls throughout this study (Figure 3A).

Next, the ability of *M. chimaera* to form biofilms on both stainless steel and titanium (data not shown) surfaces in 7H9 medium (Supplementary Figure 1, top panel) or autoclaved tap water (Supplementary Figure 1, bottom panel) to mimic the HCD environment was evaluated using FE-SEM. After 2 weeks of incubation, *M. chimaera* that was grown in both 7H9 medium and autoclaved tap water showed adhesion and robust biofilm formation on stainless steel (Supplementary Figure 1) and titanium (data not shown). The high-resolution FE-SEM imaging also revealed evidence of secretion of a mucoid EPS-like structural material on cell surfaces with appearance of appendages from *M. chimaera* cells (Supplementary Figure 1, top panel arrow). Furthermore, aggregation of *M. chimaera* cells appeared to be attached to each other end-to-end or side-to-side, to form cell clusters (Supplementary Figure 1). The individual *M. chimaera* cells that were not bound by neighboring cells showed a smaller rod-like bacilli shape. Similar to the biofilm that was observed on the polystyrene surface, *M. chimaera* cells grown on both stainless steel and titanium (data not shown) surfaces, appeared to be settled within an extracellular material. The *M. chimaera* cells embedded in the extracellular matrix may play a role in protecting cells from disinfectants and antimicrobial agents.

### Cryo-Scanning Electron Microscope (Cryo-SEM) Analysis Identifies Ultra-Structures Within *M. chimaera* Biofilm

**FIGURE 4:**
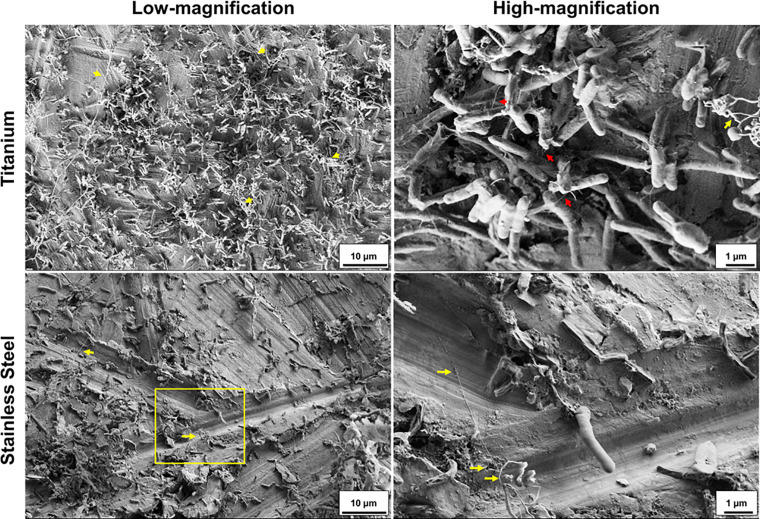
*Mycobacterium chimaera* forms a well-developed biofilm on titanium and stainless-steel surface after 2 weeks of incubation in autoclaved tap water. Representative Cryo-SEM images in low magnification (5,000: bar 10 μm) and high magnification [yellow boxed region in low magnification image: 30,000 **(top right)** or 20,000 **(bottom left)**: bar 1 μm]. *M. chimaera* (DSM 44623) biofilm on titanium surface or *M. chimaera* (2016-20-02) biofilm on stainless steel surface grown in autoclaved tap water at 30°C for 2 weeks. Ultrastructure components of *M. chimaera* Biofilm included appearance of nanowire-like structural projections (red arrow). Some of these mesh-like ultrastructural components were cryopreserved in their 3D-morphology (yellow arrow).

Next, cryo-SEM was used to visualize *M. chimaera* biofilm in a form closer to its native state with the ultra-structures and biofilm components preserved within the biofilm. *M. chimaera* biofilm grown in autoclaved tap water for 2 weeks on both titanium (Figure 4, top panel) and stainless steel (Figure 4, bottom panel) showed bacterial cells attached to each other with a network of projections that resembled bacterial nanowires (red arrows) (Leung et al., 2013). These structures appeared as a mesh of entwined strings in 3-D morphology extending throughout the biofilm matrix, between bacterial cells as well as contacting the titanium and stainless steel surfaces (Figure 4, yellow arrow).

**FIGURE 5:**
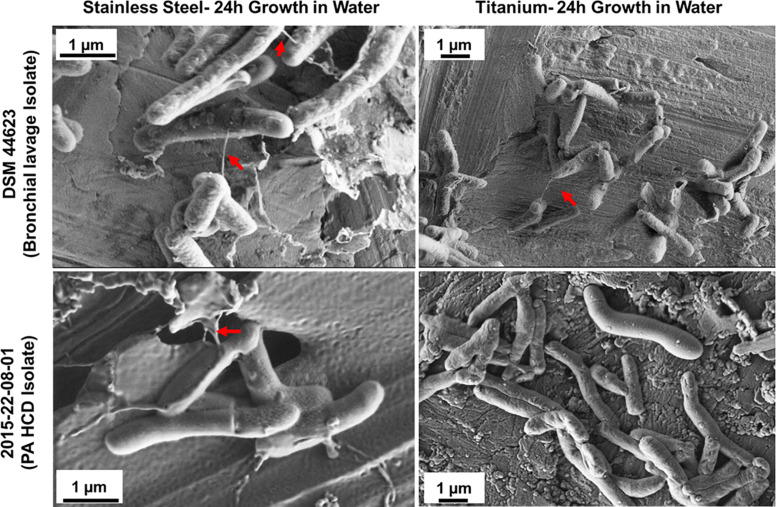
Cryo-SEM analysis reveals ultra-structures within *M. chimaera* biofilms. Three *M. chimaera* strains, DSM 44623, 2015-22-08-01 (PA HCD isolate) and 2016-20-02 (ND HCD isolate) were grown on stainless steel or titanium coupons for 24 h in autoclaved tap water. Ultra-structures were observed within these biofilms that resembled bacterial nanowire-like components (arrows).

To test the hypothesis that *M. chimaera* adhere to surfaces, particularly stainless steel, at early stage of colonization despite slow-growing nature of this bacterium – two *M. chimaera* clinical isolates, including a HCD isolates (2015-22-08-01) obtained from CDC were separately incubated for 24 h in sterile tap water on both stainless steel and titanium coupons at 30°C, and then rapidly frozen and cryo-fixed by plunging in liquid nitrogen. Cryo-SEM was then performed to visualize these biofilms on both stainless steel and titanium surfaces. Surprisingly, within 24 h, bacterial cells from both clinical isolates of *M. chimaera* attached to both stainless steel (Figure 5, left panel) or titanium surfaces (Figure 5, right panel). Cryo-SEM imaging revealed the presence of the same ultrastructures described above, seen to connect between *M. chimaera* cells within 24 h of initial inoculation, regardless of the surface or strain tested (Figure 5, arrows).

## Discussion

Bacterial biofilms play a significant role in infections associated with medical device use (Wang et al., 2017). Microbial adhesion and colonization on re-usable medical devices such as HCDs lead to the formation of biofilms that are resistant to conventional disinfection strategies (Phillips et al., 2015). HCDs colonized with microbes can serve as an ongoing source of infections in patients (Achermann et al., 2013; Sommerstein et al., 2016, 2017, 2018; FDA, 2020c). For example, HCDs colonized with *M. chimaera*, a slow-growing NTM species, has been associated with invasive infections in patients who underwent certain surgeries (CDC, 2015; Sax et al., 2015). These infections include prosthetic valve endocarditis, aortic vascular graft infections and other life-threating conditions (Achermann et al., 2013; Kohler et al., 2015; Sax et al., 2015; Chand et al., 2017; Asadi et al., 2018). To treat these infections, a prolonged antibiotic treatment and removal and replacement of infected materials was necessary, highlighting the detrimental effects of *M. chimaera* biofilm formation *in vivo* (Kohler et al., 2015; Asadi et al., 2018).

The number of publications on *M. chimaera* related case reports has increased in recent years, emphasizing the importance of eradicating *M. chimaera* infections. However, most of these studies are either clinical case reports or phylogenetic analysis with few bench research investigations on *M. chimaera*. This study provides a fundamental understanding of biofilm formation of *M. chimaera* on various medical materials which will contribute to improving the knowledge necessary for developing successful eradication methodologies for these biofilms. To our knowledge, this the first in the literature characterizing *M. chimaera* biofilm formation on medically relevant materials.

Biofilm formation is a complex multi-step process involving (1). An attachment phase where the interaction of bacteria and abiotic or biotic surfaces occurs followed by (2). Microcolony formation by cell–cell interaction and secretion of EPS matrix leading to (3). Biofilm maturation stage where modification and maintenance of the three-dimensional structure of the biofilm occur followed by the last stage of a biofilm formation (4). Detachment and dispersion, whereby bacterial cells are released from the biofilm (Costerton, 1995; Costerton et al., 1995). Bacterial attachment to different surfaces is regulated by the interplay between multiple biological factors as well as the bacterial response to surface chemistry and topography (Filipovic et al., 2020). The extracellular matrix is the “hallmark” of bacterial biofilms and accounts for 85% of the overall biofilm volume as compared to the cells constituting only 15% of the biofilm by volume (Donlan and Costerton, 2002; Flemming and Wingender, 2010). Mycobacterial biofilms are comprised of a tightly bound bacterial community encased in an extracellular matrix demonstrated to contain a substantial amount free mycolic acid (Ojha et al., 2005, 2008), glycopeptidolipids (Recht and Kolter, 2001; Howard et al., 2006), polysaccharides including cellulose (Trivedi et al., 2016), proteins (Ramsugit et al., 2013; Trivedi et al., 2016) and extracellular DNA (e-DNA)(Aung et al., 2016).

This study found that *M. chimaera* formed a robust biofilm at the air-liquid interface in borosilicate glass (Figure 1A) within 6 weeks of incubation. The air-liquid interface biofilm formation is seen other mycobacterial species and is believed to be due to microcolony aggregation and pellicle formation at the air-liquid interface of the liquid culture (Ojha et al., 2008; Totani et al., 2017). Some bacteria employ air-liquid interface biofilms to samples air while accessing nutrients from the liquid medium. So far, there are three biofilm models in use for *Mycobacteria*, including one for studying pellicle biofilms similar to the one investigated here (Totani et al., 2017; Chakraborty and Kumar, 2019). The glycopeptidolipids—monomeromycolyl-diacylglycerol—in addition to keto-mycolic acids are essential for pellicle formation as they enhance the hydrophobic nature of mycobacterial cell wall enabling cell–cell interaction in *M. avium*, a MAC complex NTM species similar to *M. chimaera* (Totani et al., 2017).

This study also quantified and compared *M. chimaera* biofilm accumulation between week 3 and week 6 of inoculation on polystyrene peg surfaces (Figure 1B). *M. chimaera* produced significantly more biofilm mass by week 6 of inoculation as compared to week 3 (Figure 1B) demonstrating that *M. chimaera* biofilm can accumulated over several weeks. Electron microscopy (FE-SEM) provided further evidence that *M. chimaera* cells on polystyrene peg surfaces are embedded in a significant amount of extracellular material by week 6 of inoculation (Figure 3). As the biofilm matured, the amount of secreted extracellular material was enhanced and may augment chemical and mechanical properties that protect NTM cells such as surface adhesive strength and cohesiveness (Hwang et al., 2014; Peterson et al., 2015). Mycolic acid, a major component of NTM extracellular biofilm matrix (Ojha et al., 2005, 2008), was also found in the *M. chimaera* EPS material (J. Amarasinghe unpublished *MALDI*-*TOF* data), indicating its potential role in biofilm formation.

The 3D Laser Scanning Microscopy images showed the ability of *M. chimaera* and *M. fortuitum* to form robust biofilms on stainless steel, titanium, silicone and polystyrene surfaces within a week of inoculation (Figure 2). Stainless steel and titanium are two of the most commonly used metals in medical devices (FDA, 2020b). Particularly, the water tanks of the HCDs are made of stainless steel (Walker et al., 2017). In addition, titanium surfaces are frequently found in surgical implants (Harris et al., 2015; FDA, 2020b) while silicon materials are used in a variety of medical devices including breast, cardiovascular, joint and ocular implants (FDA, 2019). Polystyrene plastics are present in many medical supplies and used in the current study as adhesion substratum that has already been used to quantify bacterial biofilm formation by others (Pang et al., 2012; Amarasinghe et al., 2013). The ability of these NTM to form biofilms on these medically relevant materials may be important in their role in medical device associated infections. NTM colonization has been found on HCD surfaces, aortic grafts, breast implants as well as on other medical devices (FDA, 2015, 2020a; Kohler et al., 2015; Rüegg et al., 2015; Saeed et al., 2016; Romero et al., 2017). This unexpectedly early biofilm formation by *M. chimaera* also suggests that disinfection regimens may need to be re-examined with respect to the time intervals between treatment. It is also interesting that unlike *M. fortuitum*, *M. chimaera* showed a preference for biofilm formation on metals (Figure 2, middle panel vs. right panel), which compose the largest surface area of HCDs. The preferential biofilm formation by *M. chimaera* on metal surfaces also suggests that the need for diverse strategies incorporating anti-biofilm technologies to repel microbial adhesion to eliminate the chase of microbial buildup on these medical devices.

In this investigation, the cryo-SEM technique was also performed in addition to conventional SEM, to visualize *M. chimaera* biofilm in a form closer to its native state with the ultra-structures and cell components preserved within the biofilm. It is widely reported in the literature that preparation steps for conventional FE-SEM can compromise the structural integrity of the native state of biofilm (Wu et al., 2014; Liang et al., 2019). For example, the dehydration step during sample preparation can lead to distortion of the bacterial extracellular matrix and can introduce dramatic artifacts (Azeredo et al., 2017; Liang et al., 2019). The cryo-SEM images revealed surface ultrastructure and fiber-like projections extending between cells in early *M. chimaera* biofilms of 24 h and throughout the biofilm by week 2 (Figures 4, 5). These string-like structural projections were present in biofilms of all *M. chimaera* strains tested in both stainless steel and titanium surfaces in this study indicating that this is a common behavior in *M. chimaera* biofilms regardless of the strain tested or surface used. These extracellular appendages resembled bacterial nanowires that have been previously identified in other bacterial biofilms suggesting possible importance in development and survival of *M. chimaera* biofilms (Steidl et al., 2016; Reguera, 2018). In the oral cavity, nanowires have been hypothesized to act as conductive structures in pathogenic biofilms (Rabaey et al., 2010; Wanger et al., 2013). Further investigation is necessary to determine the function of these structural projections.

Furthermore, the current study confirms *M. chimaera* to be motile in water using a light microscope, despite earlier reports stating it to be non-motile (see Supplementary Video 1). It is possible that *M. chimaera* could transition between motile and sessile mode of growth based on environmental cues, as shown in other bacterial species known to express pathogenicity *in vivo* (Morgan et al., 2006; Desai and Kenney, 2019; Desai et al., 2019). The fact that *M. chimaera* showed motility may also be related to their survival and biofilm-forming ability in HCDs and other water-bearing medical devices as motility is believed to support the initial interaction between microbes and the surface by promoting adhesion (Trautner and Darouiche, 2004; Lemon et al., 2007).

Previous studies have shown that NTM, as slow-growing oligotrophs, can survive on scarce organic matter found in drinking water (George et al., 1980; Norton et al., 2004) and in oxygen levels as low as 6% (Lewis and Falkinham, 2015). Low levels of organic matter and oxygen may better represent HCDs when not in use. While the slow growth of *M. chimaera* is thought to be a survival strategy for adapting to a nutrient-starved environment such as domestic water supplies, this may also be an advantage in resisting disinfection which is enhanced by the formation of biofilm. Metabolically dormant bacteria in biofilms have been shown to develop biofilm persisters with enhanced antimicrobial tolerance (Wood et al., 2013; Gray et al., 2019). Further work is warranted to examine the synergy between *M. chimaera* biofilm and dormancy allow it to escape conventional disinfection strategies and remerge after a period of no detectable growth (Schreiber et al., 2016; Falkinham, 2020). Given the observation that *M*. *chimaera* genome contains a plethora of diguanylate cyclase (DGC) and phosphodiesterase (PDE) genes encoding proteins with various sensory and regulatory input domains that are known to regulate biofilm formation in response to environmental cues and stress signals, biofilm regulation in *M. chimaera* is a complex process as seen in other bacteria (Amarasinghe et al., 2013; Jenal et al., 2017).

## Conclusion

Surface colonization and biofilm formation is a life stage of bacteria found throughout nature. Medical research is increasingly finding that many of the benefits that microbes derive from biofilm in the environment can also play a critical role in how microbes infect humans and resist antimicrobial treatment. To address the role of biofilms in human infection, it is important to better understand the physicochemical properties and biological mechanisms of biofilm communities in medical environments.

The challenge of biofilm is especially pernicious for NTM because of their unique hydrophobic membrane constituents—and is likely compounded by their slow metabolism, which increases the potential for dormant persister cells. The differences between NTM species can be quite significant, and likely impacts where they have a niche and thus where they are most likely to present a public health threat. For example, certain NTM have long been known to colonize plumbing, and biofilm formation by *Mycobacterium avium*, *Mycobacterium intracellulare*, and *Mycobacterium abscessus* on household plumbing materials has previously been studied (Falkinham, 2011; Mullis and Falkinham, 2013). Other NTM are known to colonize medical device implants (Rüegg et al., 2015; Romero et al., 2017) and water lines (Dutil et al., 2007; Peralta et al., 2016).

More recently, *M. chimaera*, has emerged as a source of infections specifically associated with HCD. In this work, we hypothesized that *M. chimaera*, similar to other NTM species, is adept at biofilm formation on certain medical device materials, and especially those with large surface area found in HCD such as stainless steel. The investigation of *M. chimaera* colonization in this work—in particular high resolution microscopic imaging—also contributes to the understanding of NTM biofilm formation in general. By comparing *M. fortuitum* and *M. chimaera* colonization of several common medical device materials, we discovered that there are important differences in the material preference for biofilm formation between these two species. While we did not perform competitive colonization studies between *M. fortuitum* and *M. chimaera*, it is especially interesting to note the preference for biofilm formation by *M. chimaera* on metals, which compose the largest surface area of HCD. It is important to note that the amount of biofilm formation in itself does not tell us how resistant that biofilm might be to disinfection. In past studies of HCDs that are no longer in use, *M. chimaera* was the predominant NTM species found despite the more rapid accumulation and growth of *M. fortuitum* biofilms observed in our study (Schreiber et al., 2016). Further analysis of biological mechanisms for resistance (in particular, dormancy and persister cells), and biofilm composition is needed to understand why *M. chimaera* is so persistent.

This work also reports novel features of *M. chimaera* that may be of importance in developing countermeasures. While *M. chimaera* is generally thought to be non-motile (Tortoli et al., 2004), we observed that it is motile during the process of surface adhesion and colonization. This is important because motility generally enables bacterial cells to approach preferential surfaces and is involved in both reversible and irreversible adhesion, cell migration inside microcolonies within the biofilm and subsequent dispersion of the biofilm (Tolker-Nielsen et al., 2000; Lemon et al., 2007). It is well known that bacteria have preferences for adhesion sites depending on various surface cues such as chemistry and topography. Surface topology can act as a barrier against bacterial motility and their surface exploration, ultimately impeding the biofilm formation (Chang et al., 2018). This opens the door to the possibility of coatings, changes in materials and surface patterning as a way to discourage *M. chimaera* colonization of HCD. The fact that *M. chimaera* could colonize metals rapidly within 24 h is also important because it has implications for the time period between disinfection treatments. Once cells are surface adherent and start forming biofilm, they are thought to be more challenging to disinfect. Finally, in this first high-fidelity imaging of *M. chimaera* biofilm, we found unexpectedly that ultrastructures form very rapidly compared to the normal time-scale of *M. chimaera* replication. While the slow replication rate of these microbes is generally thought to indicate a slow metabolism, in particularly due to the low level of nutrients in the water bearing environment, the rapid adhesion and development of a complex biofilm contribute to our understanding of their survival in this environment. Instead of being directed at replication, the potential that they have for metabolic activity may be directed toward establishing a robust biofilm community, from which they can persist and become more entrenched while slowly accumulating low concentration organic nutrients in the system. While the role of these ultrastructures needs further investigation, based on what is known about other bacteria it is possible that they could be some type of microtubules that conduct energy or information, both of which convey community benefits to their survival (Rabaey et al., 2010; Wanger et al., 2013; Steidl et al., 2016; Reguera, 2018).

It is important to continue developing improved disinfection processes to protect patients from NTM infections. The results presented here confirm our hypothesis that *M. chimaera* are adept at colonizing various medically relevant surfaces, and they add important information about the timing, adhesion and extent of biofilm formation, which can be exploited in the design of decontamination strategies for reusable devices such as HCD.

## Data Availability Statement

The raw data supporting the conclusions of this article will be made available by the authors, without undue reservation, to any qualified researcher.

## Author Contributions

AS, SZ, and JA designed the investigation, prepared the samples, and performed the experiments. AS and SZ performed growth assays. AS and JA performed crystal violet staining assays. AS and JA prepared samples for FE-SEM and cryo-FE-SEM. YW and KP performed FE-SEM analysis for biofilm PEGS. AS and JA drafted the manuscript with helpful suggestions from all. JA, MS, KP, and PR provided guidance and critical manuscript edits. All authors contributed and approved the submitted version of the article.

## Disclaimer

The views expressed in this manuscript are those of the authors and should not be taken as the official view or policy of the United States Food and Drug Administration (FDA), Department of Health and Human Services, or any component of the United States Government. The reference of trade names, commercial products, or organizations is for clarification of the procedures used and should not be construed as an endorsement of a product or manufacturer.

## Conflict of Interest

The authors declare that the research was conducted in the absence of any commercial or financial relationships that could be construed as a potential conflict of interest.

## Supplementary Material

Supplementary Figure 1 Field emission scanning electron microscopic images showing *Mycobacterium chimaera* forms a well-developed biofilm on stainless steel surface within 2 weeks of incubation in 7H9 medium and autoclaved tap water. Representative low magnification (5,000 or 10,000: bar 2 μm) and high magnification (yellow boxed region in low magnification image; 15,000: bar 1 μm) from FE-SEM of *M. chimaera* (DSM 44623) biofilm on stainless steel surface or control stainless steel surface without any bacteria. FE-SEM showed structural EPS-like material and appearance of surface appendages from *M. chimaera* cells (arrow).

Supplementary Video 1 *Mycobacterium chimaera* grown in autoclaved tap water in 6-well cell culture plate at 30°C for a week was video recorded using Keyence BZX 800 microscope to show motility.
