# Supplementary figures and images for: Characterization of Biofilm Formation by Mycobacterium chimaera on Medical Device Materials
Source: Front Microbiol. 2021 Jan 11;11:586657. doi: 10.3389/fmicb.2020.586657 (PMC7829485; doi:10.3389/fmicb.2020.586657)

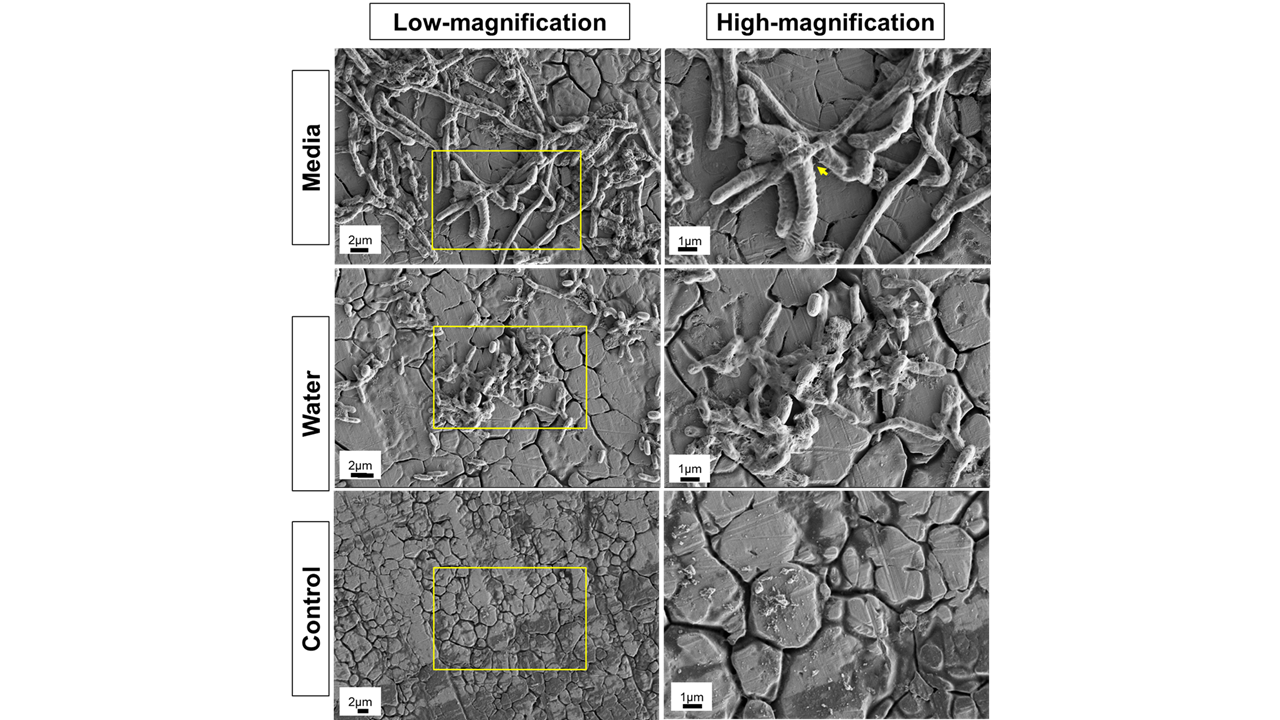

Supplement: Supplementary Figure 1 — Field emission scanning electron microscopic images showing Mycobacterium chimaera forms a well-developed biofilm on stainless steel surface within 2 weeks of incubation in 7H9 medium and autoclaved tap water. Representative low magnification (5,000 or 10,000: bar 2 μm) and high magnification (yellow boxed region in low magnification image; 15,000: bar 1 μm) from FE-SEM of M. chimaera (DSM 44623) biofilm on stainless steel surface or control stainless steel surface without any bacteria. FE-SEM showed structural EPS-like material and appearance of surface appendages from M. chimaera cells (arrow). [file Supplementary_Figure_1.tif]
